# Direct Single-Impact Electrochemistry Using Silver Nanoparticles as a “Digital” Readout for Biosensing Applications

**DOI:** 10.1021/acssensors.5c00064

**Published:** 2025-06-14

**Authors:** Sebastian Freko, Lennart J.K. Weiß, Friedrich C. Simmel, Bernhard Wolfrum

**Affiliations:** 1 Neuroelectronics, Munich Institute of Biomedical Engineering, Department of Electrical Engineering, School of Computation, Information and Technology, 9184 Technical University of Munich, Garching 85748, Germany; 2 Department of Medicine I, Cardiology, Angiology, Pneumology, Klinikum rechts der Isar, Technical University of Munich, TUM School of Medicine and Health, Munich 81675, Germany; 3 Department of Bioscience, TUM School of Natural Sciences, Technical University Munich, Garching 85748, Germany

**Keywords:** single-impact electrochemistry, nanoimpact electrochemistry, single-entity electrochemistry, stochastic collision electrochemistry, silver nanoparticles, biosensing, point-of-care, “digital” readout

## Abstract

Direct single-impact electrochemistry is a rapidly evolving analytical method based on the collision of redox-active species, such as silver nanoparticles (AgNPs), with a biased microelectrode. The collision results in distinct current spikes due to partial or complete oxidation of a particle. In recent years, this technique has been applied in various biosensing strategies as a “digital” readout technique. It offers the quantification of analytes using discrete signals, as opposed to conventional amplitude-based methods. In this review, we explore the latest advancements in direct single-impact electrochemistry for biosensing applications. In addition, we summarize the key factors influencing the “digital” readout performance and their interrelationships, including particle size and corona, electrode size and potential, electrolyte composition, particle mass transport toward the electrode, and data acquisition. Considering recent experimental developments and theoretical principles, we have identified guidelines that are expected to facilitate and accelerate the development of novel direct impact-based sensing platforms, particularly for point-of-care (POC) applications.

## Graphical abstract

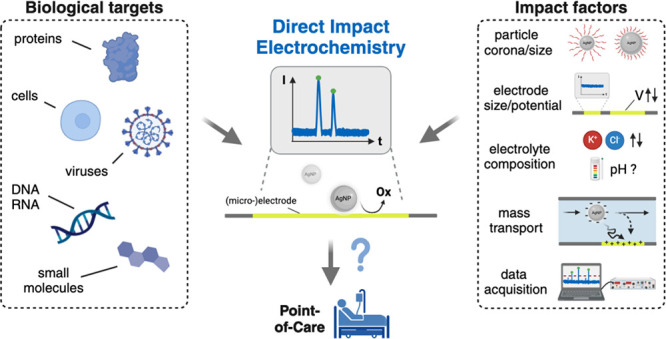

Over the last two decades, the field of single-impact electrochemistry, also referred to as stochastic impact, stochastic collision, nanoimpact, or single-entity electrochemistry, has gained increasing attention as a highly sensitive analytical method that is based on the collision of single entities with a biased electrode of μm size.^1–9^ As the current response upon impact strongly depends on the colliding species, the field can be classified into four main approaches: blocking, catalytically amplified, direct, and droplet/vesicle impacts, as schematically illustrated in Figure 1. The blocking impact approach was pioneered by Lemay and co-workers in 2004, as they demonstrated the detection of micron-sized latex beads on gold microelectrodes in the presence of ferrocene methanol.^10^ The impacts of these electro-inactive beads resulted in a steplike decrease in the current response, as the beads hindered (“blocked”) the mass transfer of the redox mediator to the electrode (Figure 1a). Recently, the diffusion-blocking strategy was used for the detection and analysis of red blood cells,^11^ bacteria,^12^ human platelets,^13^ and biomacromolecules such as proteins and DNA.^14^ Three years after Lemay’s publication on blocking impacts, Bard and colleagues reported catalytically amplified impacts as they detected single platinum nanoparticles (PtNPs) on a carbon fiber ultramicroelectrode.^15,16^ In contrast to the blocking approach, the colliding PtNPs catalyze a secondary reaction, e.g., the evolution of hydrogen, leading to a steplike increase in the current, as shown in Figure 1b. To date, the electrocatalytic strategy using PtNPs has been used to detect and quantify biological samples, e.g. microRNAs.^17–22^ Furthermore, other active and low-cost catalyst, such as Co_3_O_4_, have been investigated toward their oxygen evolution reaction catalytic activity using single-impact electrochemistry.^23,24^ In 2011, Compton and co-workers reported on the direct electrooxidation of AgNPs.^25^ In this approach, the collision enables the direct conversion of the particle and yields a sharp current spike, as illustrated in Figure 1c. This type of impact has been extensively studied, in particular regarding the effects of different particle sizes,^26^ capping agents,^27–29^ variations in the electrolyte composition,^30–33^ electrode functionalization,^34,35^ and the kinetics of the electron transfer during a nanoparticle impact.^36–40^ Later, Bard’s group explored a fourth type of impact: droplet and vesicle impacts (Figure 1d).^41,42^ In these studies, toluene droplets containing ferrocene and vesicles encapsulating potassium ferrocyanide collided with the electrode surface, releasing the redox probe. Additionally, this strategy has also been used to study biological contents, including catecholamine inside individual vesicles in living pheochromocytoma cells with or without l-3,4-dihydroxy-phenylalanine^43^ and vitamin C.^44^

**Figure 1:**
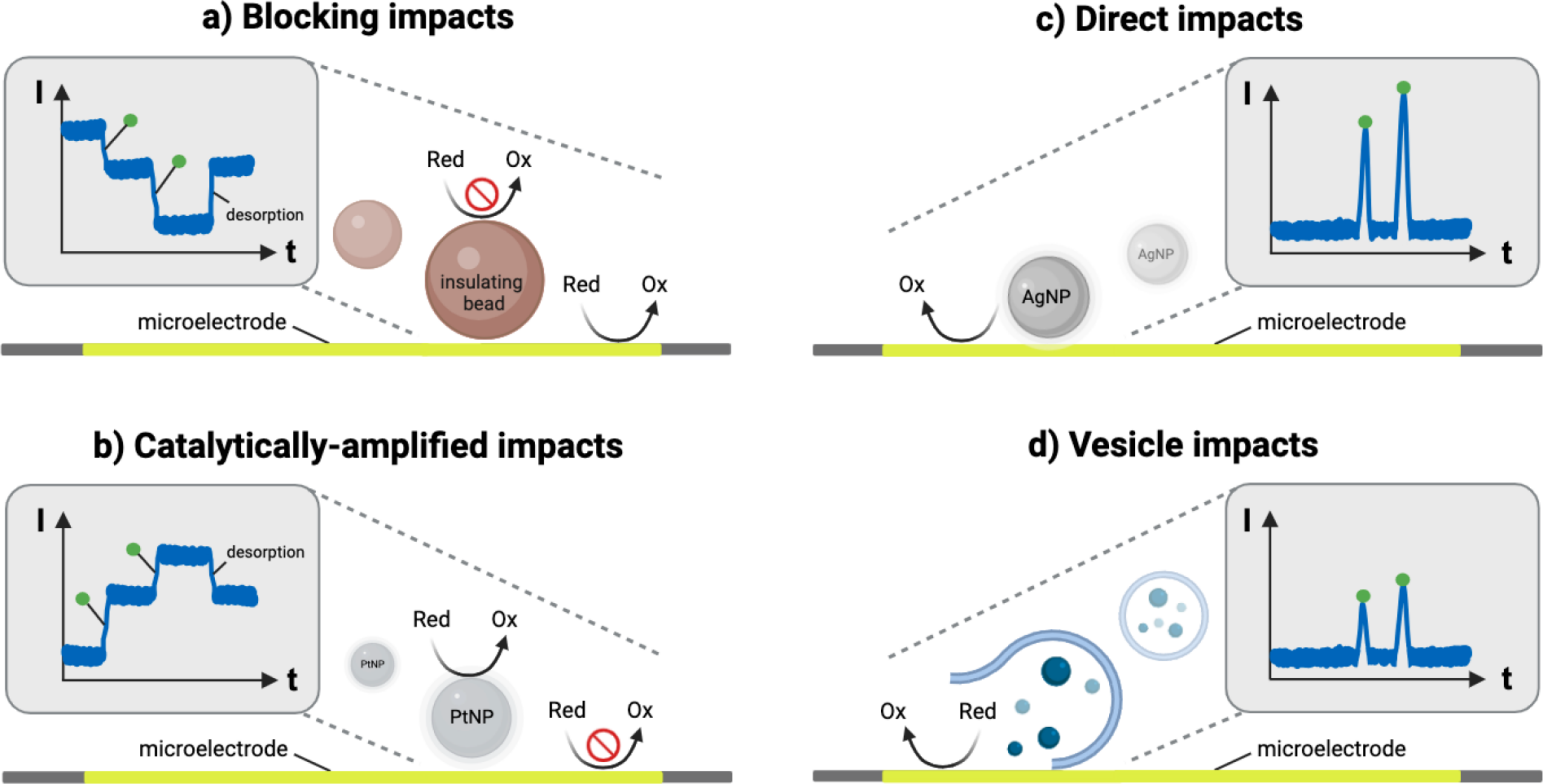
Different types of impacts in single-impact electrochemistry. (a) In a typical blocking impact experiment, an insulating species, such as latex beads, cells, or bacteria, collides with and adsorbs to the electrode, limiting the mass transport of the redox-active species from the bulk solution to the surface of the electrode. Individual impacts are observed as a steplike decrease in redox current. Desorption of the particles leads to a recovery of the current prior to the impact. (b) In contrast, catalytically active particles, such as PtNPs, generate an additional catalytic current when colliding with the electrode, resulting in a stepwise increase in current. (c) Redox-active metal nanoparticles like AgNPs collide with an appropriately biased electrode, where electrooxidation results in a distinct current peak. (d) Upon the collision of droplets and vesicles containing a redox-active species, their membrane may rupture, releasing the redox probe and giving an anodic current spike. Green dots indicate individual impacts. This figure was created with BioRender.com.

The stochastic collision of nanoparticles results in a distinct current response (“1”) compared to the background noise (“0”), which allows a binary-like quantification that can be used as a “digital” readout strategy. Herein, we focus exclusively on biosensing applications that use the direct impact approach since this method offers a high signal-to-noise ratio (SNR) and can easily be applied without further target amplification strategies.^5,45^ Assuming complete oxidation events and a spherical geometry of the nanoparticle, its radius (*r*_NP_) can be calculated based on the transferred charge (*Q*) during the impact:^46^

$$r_{\mathrm{NP}}=\sqrt[{3}]{\frac{3M_{\mathrm{NP}}Q}{4\pi zF\rho_{\mathrm{NP}}}}$$ 1

where *M*_NP_ is the molar mass, *z* is the valency of the colliding NP, *F* is Faraday’s constant, and ρ_NP_ is the density of the particle. Therefore, this method allows quantitative nanoparticle sizing, complementing common sizing techniques, such as transmission electron microscopy and dynamic light scattering.^46,47^ This method is not exclusive to AgNPs but other metal NPs, such as Au,^48^ Ni,^49,50^ and Cu,^51^ can also be oxidized using appropriate electrode materials and potentials. If we consider an experiment, where particles collide by virtue of their Brownian motion onto a disk microelectrode, with the radius *r*_el_ *,* the steady-state impact frequency *f*_0_ is directly proportional to the concentration of the particles in the bulk solution (*c*_0,NP_)­

$$f_{0}=4D_{\mathrm{NP}}N_{A}c_{0,\mathrm{NP}}r_{\mathrm{el}}\;\mathrm{with}\;D_{\mathrm{NP}}=\frac{k_{B}T}{6\pi \eta_{\mathrm{vis}}r_{\mathrm{NP}}}$$ 2

Here, *D*_NP_ represents the diffusion coefficient of a spherical particle with *r*_NP_, *N*_A_ is Avogadro’s constant, *k*_B_ *T* is the thermal energy, and η_vis_ is the viscosity of the fluid. Consequently, diffusion to the electrode, and thus *f*_0_, decreases with increasing particles sizes.^52^ The induced electro-dissolution process–characterized by the impact frequency and the amount of transferred charge–depends on several factors, including the particle corona and the electrolyte composition. For instance, a decrease in impact frequency has been observed for increasing shell thickness or increasing degree of aggregation. Such findings have provided the framework for the development of various impact-based sensing concepts that modulate the impact frequency of AgNPs depending on the target concentration. For example, such sensing concepts have been used to detect and quantify a broad range of clinically relevant targets, including nucleic acids (DNA/RNA),^45,53^ proteins,^54–56^ cells,^57^ viruses,^58^ and other molecules.^59,60^ The high SNR (impact amplitudes can be >100 times higher than the baseline current, depending on the particle size) regardless of the target concentration and the quantification using discrete signals by simply counting the impacts during a defined measurement interval offer advantages over conventional amplitude-based sensing strategies. Moreover, the first developments of a low-cost, portable nanoparticle detection device for the on-site monitoring of AgNPs demonstrated the potential of direct nanoimpact electrochemistry at the POC.^61^

In this review, we provide a comprehensive overview of recent developments in direct single-impact electrochemistry for biosensing applications, highlighting different strategies for utilizing AgNPs as a “digital” readout. In addition, we systematically summarize the detection method’s main influence factors and discuss the challenges and advances. By considering both experimental developments and theoretical principles, we outline guidelines and trends that could assist in designing novel direct impact-based sensing platforms for POC applications.

## Influence Factors on Direct Single-Impact Electrochemistry: Challenges and Advances

### Particle Size and Corona

Understanding the factors influencing the oxidation behavior of AgNPs is essential to overcome the current challenges and help translate the concept into reliable next-generation POC sensors. Generally, the spike amplitude and the transferred charge per impact increase with particle size, as indicated in Figure 2a. While 10 nm AgNP particles are oxidized in a single event, particles between 20 nm and 40 nm usually require several stripping events before complete dissolution and even larger particles (≳60 nm) tend to diffuse back to the bulk solution before complete oxidation.^26^ Consequently, increasing the size of the AgNPs leads to partial oxidation and potentially multiple peaks arising from an individual particle.^62^ Therefore, this behavior could intentionally be used to amplify the signal, particularly for concepts that are based on the release of AgNPs, as one target molecule results in several spikes.^63,64^ To enable the complete oxidation of (large) particles, the dwell time of the particles in the tunneling region must be prolonged, resulting in a more effective charge transfer and higher current amplitudes. Defnet and Zhang implemented this idea by modifying a gold electrode with a polysulfide layer using sodium thiosulfate as an electrolyte.^65^ The ultrathin sulfide layer enhanced the sticking probability of the particles, thereby enabling full oxidation for AgNPs up to 100 nm according to their hypothesis. They observed a 25-fold increase in collision frequency and a 3-fold increase in amplitude for 60 nm particles. In addition, a study from Dery et al. suggested that charged self-assembled monolayers on the electrode could improve charge transfer and support complete oxidation for larger nanoparticles.^35^ Apart from the AgNP size, the ligand type and its density substantially influence the charge transfer and, consequently, the detectability of the particles. To assess the influence of the particle corona, the oxidation of AgNPs modified with various species, including alkanethiols,^29^ DNA,^66^ and antibodies,^54,55^ has been investigated. Generally, thicker and denser shells reduce the efficiency of charge transfer and lower the spike amplitudes, as thicker coronas impede the particle′s trajectory to the tunneling region, making these particles more challenging to detect.^67^ In contrast, the functionalization of particles can also provide benefits for nanoimpact detection. For instance, functionalized particles are substantially more stable in high-salt concentrations^68^–relevant for the detection in biological samples–than unmodified AgNPs (typically citrate-capped). The latter exhibit low reported critical aggregation concentrations of ∼25 mM KCl (20 nm-sized) and ∼50 mM NaCl (70 nm-sized).^45,69,70^ Therefore, there is a crucial trade-off between particle stability and detectability, and modifications must be tailored to the specific applications.

**Figure 2:**
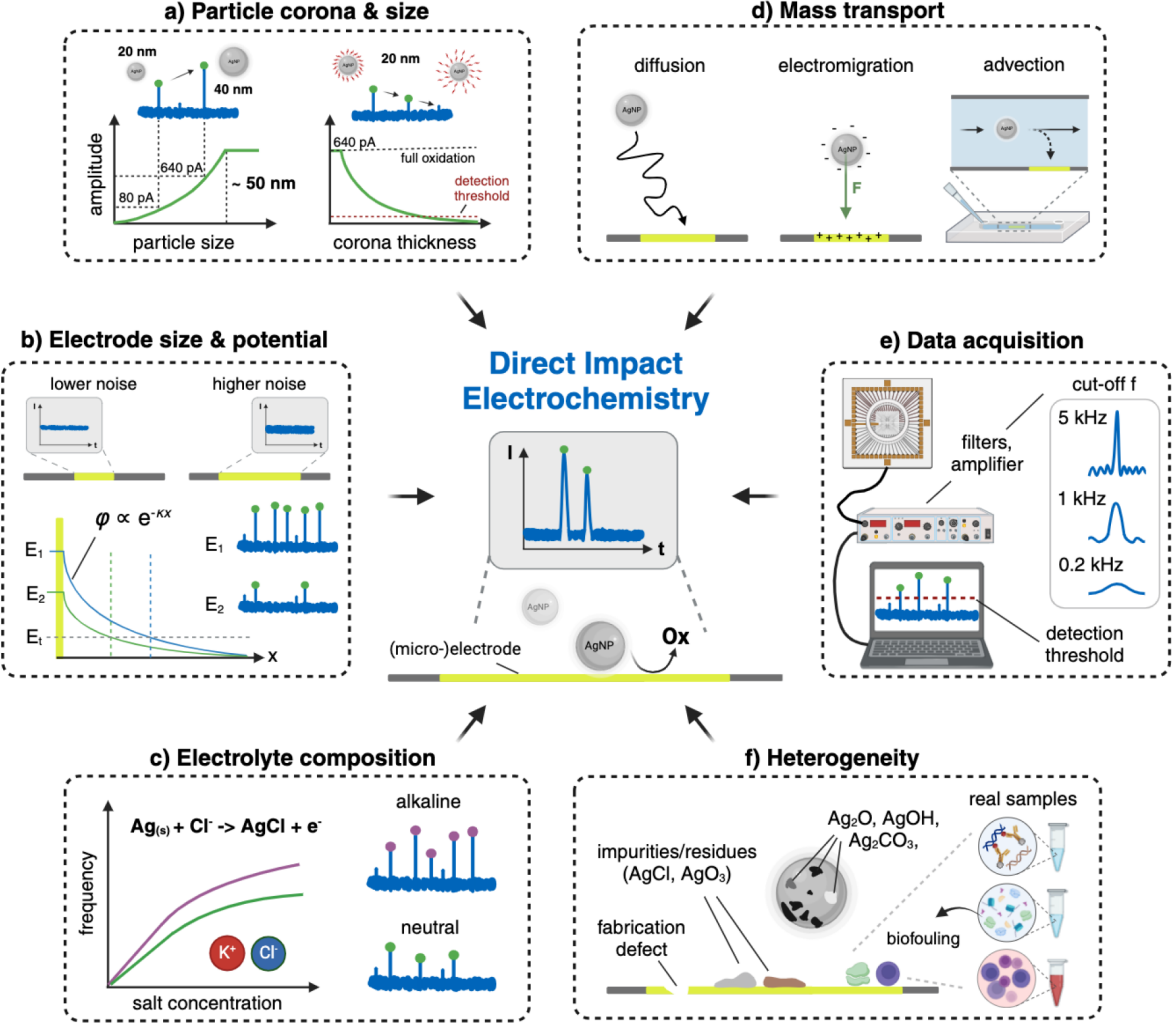
Main factors that influence direct collision impact electrochemistry include but are not limited to (a) particle size and corona. Generally, larger particles yield higher current amplitudes and transfer more charge per impact. The transferred charge per particle decreases with increasing ligand size and density. (b) Electrode size and applied potential. Smaller electrodes provide lower baseline currents to identify small current spikes. The overpotential decays into the bulk solution and is influenced by the properties of the detection solution (κ is the inverse Debye length). Increasing the overpotential expands the tunneling region, promoting higher amplitude peaks. (c) Electrolyte composition. Higher electrolyte concentrations lead to higher impact frequencies. In addition, alkaline detection conditions support higher impact rates and increased current amplitudes. (d) Mass transport, which is governed by diffusion, electromigration, and advection, is crucial for achieving high detection efficiencies within reasonable measurement times. (e) Data acquisition and data processing. The impact frequency and peak shapes are influenced by parameters such as bandwidth and the peak detection threshold. (f) Heterogeneity of the detection electrodes, AgNP surface, and (biological) samples influence the oxidation behavior. This figure was created with BioRender.com.

### Electrode Size and Potential

Traditionally, microelectrodes (5–25 μm in diameter) are used in single-impact electrochemistry, as smaller electrodes exhibit lower noise levels in an amperometric measurement due to the increased impedance. However, decreasing the electrode size reduces the likelihood of a particle colliding with the electrode.^71^ Nevertheless, sufficiently low baseline noise levels are crucial to detect the small current spikes (Figure 2b). This is particularly important when detecting smaller particles that transfer less charge and generate lower spike amplitudes, highlighting the interconnection between particle and electrode size. For instance, a 20 nm-sized particle (∼80 pA, for an approximate peak duration of ∼0.5 ms) results in a reduced SNR by a factor of ∼8 compared to a 40 nm particle (∼640 pA), assuming that the spike duration remains constant. Thus, a partially oxidized particle with a lower amplitude spike might remain undetected.

In addition to the electrode size and modifications, the applied potential plays a crucial role, as it determines the reaction dynamics.^64,72^ The electrode potential can be approximated by an exponential decay into the bulk solution according to the Gouy–Chapman model, as indicated in Figure 2b. The decay is influenced by solution properties, such as the electrolyte concentration.^63,64^ It has been observed that low overpotentials (η < 50 mV, 20 mM KCl) lead to smaller current amplitudes and prolonged peak durations, indicating impeded charge transfer, which may also result in partial oxidation events.^72^ The partial dissolution of the AgNP can lead to multiple collisions, giving secondary peaks of varying shapes, e.g., a single smaller peak, tailed peak, or multipeak, depending on the trajectory of the AgNP residue.^64^ In contrast, higher overpotentials (η > 50 mV, 20 mM KCl) accelerated the electrooxidation rate, promoting complete oxidation and producing sharper spikes with increased amplitude.^72^ Higher applied potentials might be useful to oxidize functionalized particles as they expand the tunneling region. On the other hand, they can contribute to higher noise levels and electrochemical background reactions.^73^ In addition, the applied potential is limited by the possible oxidation of the electrode material, e.g., platinum oxidation.^74,75^ In the case of higher overpotentials, the reaction rate is also limited by the diffusion of coreactant species to the particles.^30,31^ A more detailed description of how the electrolyte influences the reaction kinetics and oxidation behavior of AgNP is given in the following subsection.

### Electrolyte Composition

As we indicated in the previous section, the composition of the buffer is crucial. In a typical nanoimpact experiment with an unmodified electrode, KCl is commonly used as the detection buffer since chloride ions bind strongly to the oxidation product, forming silver chloride, according to the following reaction:^30^

$${\mathrm{Ag}}_{\left(s\right)}+{\mathrm{Cl}}_{\left(\mathrm{aq}\right)}^{-}\to \mathrm{Ag}\mathrm{Cl}+e^{-}(E^{0}=0.22\;V)$$ 3

where *E* ^0^ is the standard electrode potential versus SHE. In addition, another reaction path includes the formation of silver ions (*E* ^*0*^ = 0.80 V versus SHE). One or both reaction paths can take place depending on the electrode potential.^76^ Krause et al. showed that a minimum chloride concentration of ∼2 mM is required for reliable detection of 20 nm AgNP, with higher KCl concentrations leading to increased impact frequencies and a faster dissolution kinetics, which results in sharper current spikes.^31^ Similar reaction kinetics were also found for other halide ions, e.g., Br^–^ and I^–^ (*E* ^0^ = 0.07 and −0.15 V vs SHE for oxidation to AgBr and AgI, respectively),^30,77^ where increased concentrations led to higher impact rates, suggesting that the oxidation of AgNP is strongly dependent on the diffusion of the electrolyte species toward the particle.^30,39,78^ On the other hand, the detection in water–alcohol mixtures showed the opposite behavior. The diffusion of halide ions was reportedly hindered by the presence of larger solvent molecules, leading to broader current peaks.^79^ In addition to elevated electrolyte concentration, the detection in alkaline solutions generally yields higher impact rates and current amplitudes, as schematically depicted in Figure 2c. It has been reported that the formation of an insoluble oxide layer in alkaline solution increases the adsorptive interactions and prolongs particle dwell times.^80^ Consequently, high electrolyte concentrations and basic pH conditions are desirable for efficient detection but are limited by the aggregation kinetics of the AgNPs. Improving particle stability in high electrolyte concentrations can be achieved via functionalization, as previously discussed. However, a dense and thick corona might compromise the benefit of a higher electrolyte concentration for the detection–an essential trade-off to keep in mind during the experimental design.

Finally, as previously discussed, sodium thiosulfate has been used as an electrolyte in combination with polysulfide-modified electrodes, resulting in higher impact frequencies and particularly higher amplitude peaks.^53,57,58,65^ In this approach, it is suggested that the colliding AgNPs directly react with the polysulfide layer, forming insoluble silver sulfide, as shown in eq 4.^65^ In a subsequent step, the silver sulfide on the electrode can then react with the bulk thiosulfate to form a soluble product, as seen in reaction 5.

$$2\mathrm{Ag}+S0⇆{\mathrm{Ag}}_{2}S_{\left(s\right)}$$ 4

$${\mathrm{Ag}}_{2}S_{\left(s\right)}+4S_{2}O_{3}^{2-}⇆2\mathrm{Ag}(S_{2}O_{3}{)}_{2}^{3-}+S^{2-}$$ 5

The higher reported peak amplitudes achieved with this method may be beneficial when using larger electrodes that have a higher baseline noise. However, an additional step is required to modify the electrodes, which can be done *in situ* using a potential pulse.

### Mass Transport

In general, the mass transport to the electrode is governed by diffusion, electromigration, and advection (e.g., within supporting microfluidics), as depicted in Figure 2d. The superposition of the individual transport mechanisms results in a combined flux (density) $\vec{j}$ that can be described via

$$\vec{j}={\vec{j}}_{\mathrm{diff}}+{\vec{j}}_{\mathrm{em}}+{\vec{j}}_{\mathrm{adv}}=-D_{\mathrm{NP}}\nabla c_{\mathrm{NP}}+({\vec{u}}_{\mathrm{ep}}+{\vec{u}}_{\mathrm{eo}}+{\vec{u}}_{m})c_{\mathrm{NP}}$$ 6

where ${\vec{u}}_{\mathrm{ep}}$ describes the electrophoretic particle velocity, and ${\vec{u}}_{\mathrm{eo}}$ and ${\vec{u}}_{m}$ are the particle velocities caused by electroosmosis and externally induced flow, respectively. As discussed, moderate to high electrolyte concentrations, depending on the colloidal stability of the (functionalized) AgNPs, are preferably used in nanoimpact experiments, leading to a Debye length (λ_D_) in the order of a nanometer.^81^ In addition, the small size of the systems and minor changes in the density of the detection solution during the experiments do not introduce considerable advection. For diffusion-dominated systems, eq 6 simplifies to Fick’s first law ( ${\vec{j}}_{\mathrm{diff}}=‐D_{\mathrm{NP}}\nabla c_{\mathrm{NP}}$ ). However, concepts purely relying on diffusion for mass transport of particles result in long detection times for low concentrations due to a small *D*_NP_ of AgNPs (∼10^–11^ m^2^/s).^81^ For example, assuming a diffusive steady-state flux of 20 nm particles to a 10 μm diameter electrode (eq 2) it would take, on average, more than 5 min to observe a single impact for a concentration of 10 fM. For electrophoretic transport, in most realistic scenarios’ the condition *r*_NP_ ≫ λ_D_ is satisfied, and the electrophoretic mobility then obeys the Helmholtz-Smoluchowski equation

$$u_{\mathrm{ep}}=\frac{\varepsilon \zeta_{\mathrm{NP}}}{\eta_{\mathrm{vis}}}$$ 7

where ε is the permittivity of the solution and ζ_NP_ the zeta potential of the particle.^52^ Thus, migration plays a crucial role for all particle sizes and shapes and can even dominate over diffusion at typical electrolyte detection conditions for larger particles (*r*_NP_ ≳ 50 nm) with low *D*_NP_.^52^ Therefore, more recent detection schemes try to leverage further mechanisms to improve the mass transport to the electrode, as it is still a major limitation of the surface-based detection approach. In practice, electromigration affects particles, as an electric field is often present due to background currents occurring at the working electrode.^46,52^ Consequently, electromigration can be used to attract particles or repel them depending on the direction of the electric field.^52^ As an example, the use of on-chip electrokinetic micropumping has been demonstrated.^82^ By implementing a macroscopic electrode that surrounded an array of microelectrodes for the detection, the particle trajectories could be modulated by electroosmosis and electrophoretic migration. In addition, supporting microfluidics has been used to enhance particle mass transport toward the electrode.^83^ Furthermore, a substantial increase in the impact frequency and the detection of previously dried AgNPs to mimic a lateral flow sensor, was achieved by surface-controlled advection using a paper-based microfluidics, demonstrating the potential for POC applications.^84^ Apart from long detection times, significant adsorption particularly of unfunctionalized AgNPs at the passivation (insulating sheath) of the electrode can occur, resulting in a lower impact frequency.^85^ Here, electromigration can be used to counteract this effect. By introducing a negatively biased shield electrode in proximity to the detection electrodes, negatively charged AgNPs are electrostatically repelled, and fewer particles form the bulk adsorb at a passive interface.^70^

Overall, electromigration and advection can be used to improve the detection efficiency. Different strategies could include mixing structures,^86^ three-dimensional electrode arrays fabricated using additive manufacturing (possibly combined with microfluidics),^87^ and high-density electrode arrays based on complementary metal oxide semiconductor systems.^88^ Furthermore, cylindrical microwire electrodes can be used to increase the collision probability.^89^ Achieving high detection efficiencies is crucial to quantify very low AgNP concentrations in a reasonable time, e.g., in environmental sensing to probe contaminated water. To enhance detection capabilities in biosensing, current concepts are often coupled with enzymatic amplification strategies, such as CRISPR/Cas or DNAzyme-based amplification.^45,57^ In these approaches, direct single-impact electrochemistry is used as a “digital” readout to quantify a target analyte by measuring an impact frequency and correlating it to a present target concentration using a calibration curve.

### Data Acquisition and Processing

A challenging aspect of direct collision electrochemistry is the accurate acquisition of data. A typical experimental setup is illustrated in Figure 2e. It includes an array of detection electrodes, a suitable low-noise amplifier system, and a spike-detection algorithm. Since the current amplitudes of the impacts are usually low (picoampere) and the time scales fast (micro- to milliseconds), the requirements for the detection electronics become challenging for decreasing particle sizes.^8^ Therefore, a commonly used strategy to record the low and fast current transients is to increase the SNR and ensure low background noise. To this end, transimpedance amplifiers with feedback resistors in the GΩ range are combined with strong low-pass filtering.^90^ The reduction in bandwidth has the additional advantage that lower sampling rates are required for the analog-to-digital conversion. However, applying a low-pass filter decreases the time resolution, effectively limiting the number of well-resolvable spikes within a given time window. As a result, fast consecutive impacts may be merged into a single spike.^91,92^ Additionally, limiting the bandwidth substantially distorts the signal, leading to peak broadening and lower spike amplitudes, as shown in Figure 2e.^90^ Short-duration signals from nanoimpacts, however, exhibit a broad frequency spectrum, including high-frequency components. Therefore, a sufficiently high bandwidth of the filter and the amplifier is crucial to preserve the pulse shape, amplitude, and duration of short impact signals, whereas the charge of the impact is generally conserved.^90,91,93^ To enable recordings on larger detection areas without merging fast successive spikes or compromising noise levels, microelectrode arrays (MEAs) that have a number of individually addressable channels (*N*_c_) can be used. The detection time to obtain the same number of impacts compared to a single microelectrode is then reduced by $\frac{1}{N_{c}}$ , assuming that the electrodes are spaced sufficiently far away to not compete for the particle flux. Further strategies to improve the SNR include specialized Faraday cages and self-adaptive filters, such as Kalman filter and wavelet transform denoising.^90,94^

### Heterogeneity and Reproducibility

Apart from the discussed influence factors, inherent challenges associated with electrode fabrication, particle aging, and real sample testing impact the sensing performance, particularly its reproducibility (Figure 2f). For instance, microfabrication defects, such as microcracks or pinholes, lead to variances in the performance of individual electrodes and electrode arrays. This could impact the experiment’s outcome, especially when measuring low concentrations or small absolute changes in impact frequency. Additionally, impact experiments have been shown to be sensitive to electrode impurities,^95^ a problem that is even more pronounced when the detection electrodes are reused multiple times. In this case, appropriate electrochemical cleaning is beneficial to remove previously deposited silver chloride or other silver oxide residues.^82^ Impurities or modifications on the particle surface also influence the impact efficiency. For example, a silver oxide layer on the particle surface in alkaline media can lead to a symmetric or asymmetric electro-dissolution, depending on its surface distribution (Figure 2f).^96^ In addition to an oxide layer, AgNPs may have hydroxide, oxyhydride, or carbonate layers that affect their redox activity.^96^

Finally, measuring biological targets in complex media (e.g., human serum or blood) using an electrochemical readout presents additional challenges, such as electrode fouling, which refers to the undesired accumulation of material on the surface of the electrode.^97^ Biofouling, specifically, involves the adsorption of biomolecules, such as proteins or lipids, reducing the electroactive surface and impairing the electrochemical performance.^98,99^ Zhou and co-workers observed inhibition of electron transfer due to electrochemically inactive species to detect alpha-fetoprotein in real serum samples using direct nanoimpacts. They addressed this issue by diluting the samples 200-fold.^55^ On the contrary, Guo et al. reported no difference between the impact rates in buffer compared to human serum that was only diluted 10-fold.^53^ In general, heterogeneity of the electrode quality, the electrochemical properties of the AgNPs, and the adsorption of unwanted biomolecules influence reproducibility and should be addressed to enable reliable “digital” biosensors based on direct collision electrochemistry. To minimize aging effects, the particles should be stored under the same (potentially inert) conditions. Furthermore, to decrease the influence of previous experiments, single-use chips might be beneficial compared to reusable chips. Lastly, larger electrodes in combination with larger particles could offer a more reproducible readout, as small defects or impurities on the electrode or particle surface generally have a smaller effect on the overall signal.

## Direct Single-Impact Electrochemistry in Biosensing Applications: Advances and Future Directions

AgNPs are widely used in direct single-impact electrochemistry due to their fast oxidation kinetics upon collision with an appropriately biased (micro)­electrode. Here, we discuss general biosensing strategies on how AgNPs can be exploited as a “digital” readout in direct single-impact electrochemical detection. The first class involves the capture and release of (functionalized) AgNPs upon exposure to target molecules, as exemplarily shown in Figure 3. The second class includes the aggregation and deaggregation of AgNPs in the presence of the target, modulating the impact frequency during the detection (Figure 4)**.** A further approach relies on the modulation of the charge transfer during particle impacts in the presence of the target and is schematically depicted in Figure 5. Finally, we summarize recent applications utilizing specific targets (Table 1).

**Figure 3:**
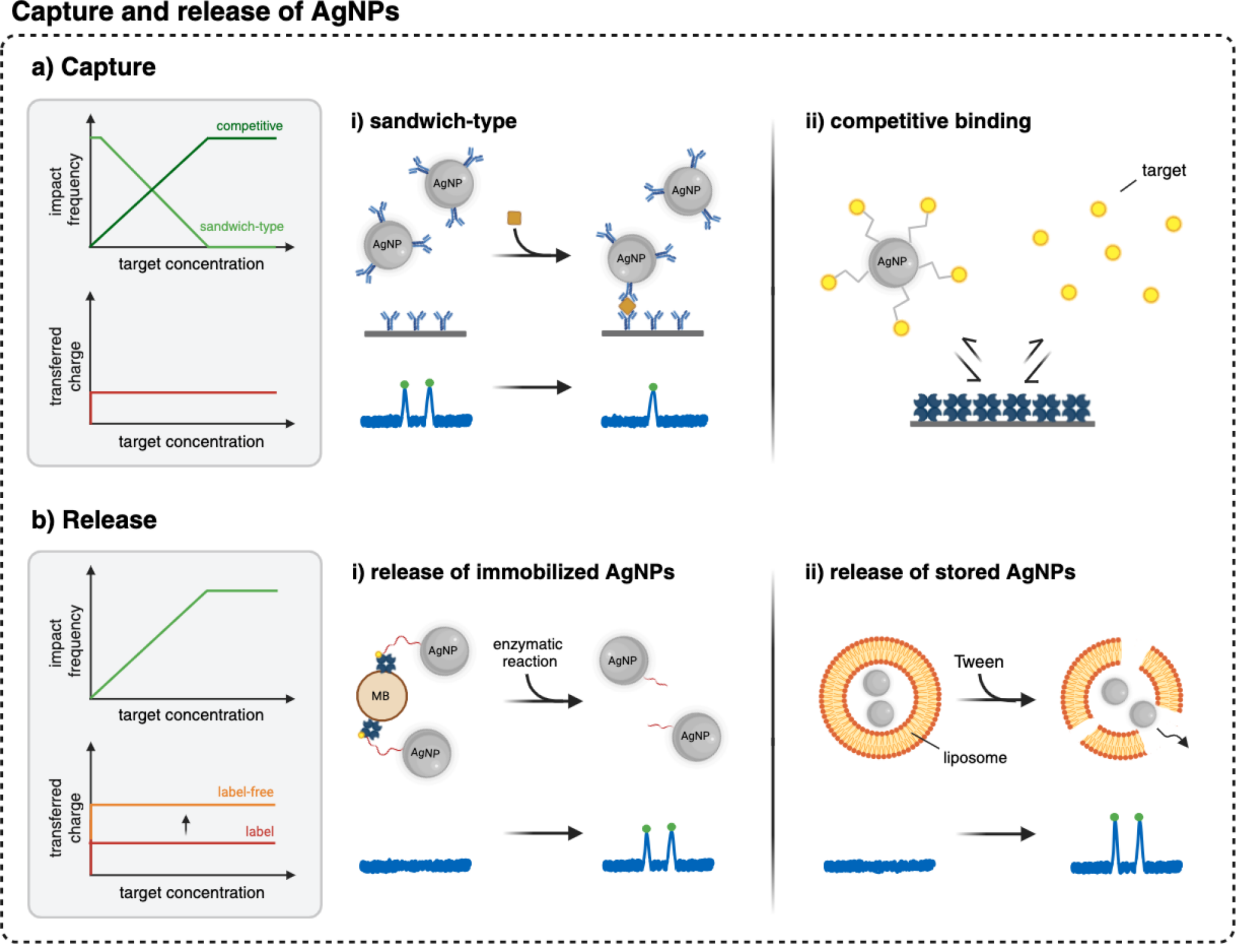
Representative implemented and potential strategies for employing AgNPs in biosensing applications using capture and release mechanisms. (a) Capture of functionalized AgNPs. (i) Sandwich-type assay, in which antibody-labeled AgNPs are captured on immobilized antibodies in the presence of the target. Unbound AgNPs can be detected, and the impact frequency decreases with increased target concentration. (ii) Competitive binding assay. AgNPs functionalized with the target molecule competing with the free target molecules. The higher the free target concentration, the less functionalized AgNPs are captured–consequently, the impact frequency increases. The charge and peak current are independent of the target concentration. (b) Release of (i) preimmobilized AgNPs from magnetic beads using an activated Cas enzyme and (ii) stored AgNPs encapsulated in liposomes using Tween. Impact frequency increases with target concentration. The transferred charge per impact is independent of the target concentration, and the label-free strategy exhibits higher charge transfer and higher peak currents. The *x*-axis (target concentration) often represents a logarithmic scale. This figure was created with BioRender.com.

**Figure 4:**
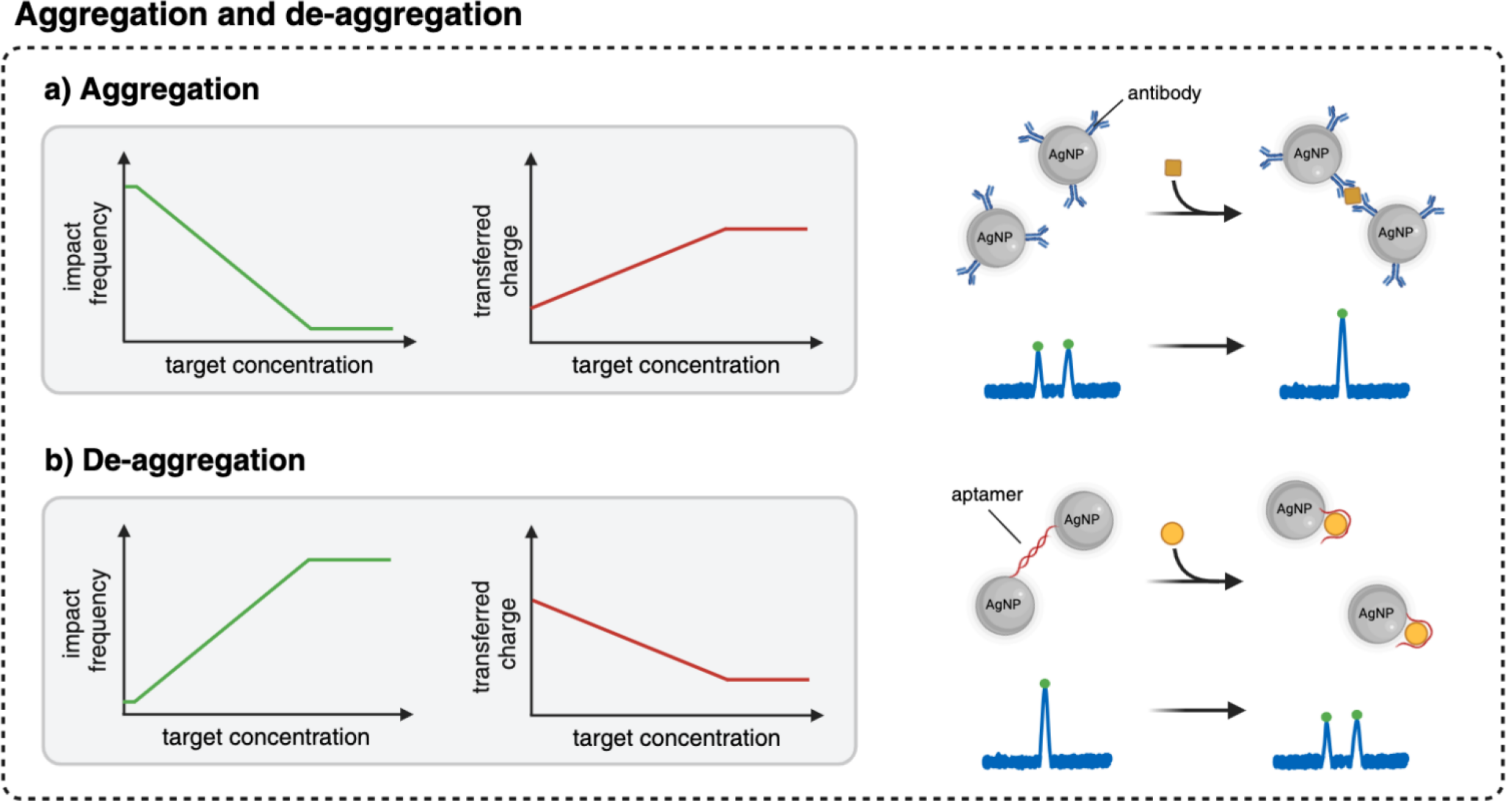
Representative strategies for employing AgNPs in biosensing applications using aggregation and deaggregation. (a) Aggregation of antibody-labeled AgNPs in the presence of target proteins, leading to altered impact characteristics, such as a decreased impact frequency and an increase in charge transfer per impact. (b) Deaggregation of reversibly aggregated AgNPs due to partially complementary aptamers increases the impact frequency upon target binding to the aptamers and decreases the charge transfer. The *x*-axis (target concentration) often represents a logarithmic scale. This figure was created with BioRender.com.

**Figure 5:**
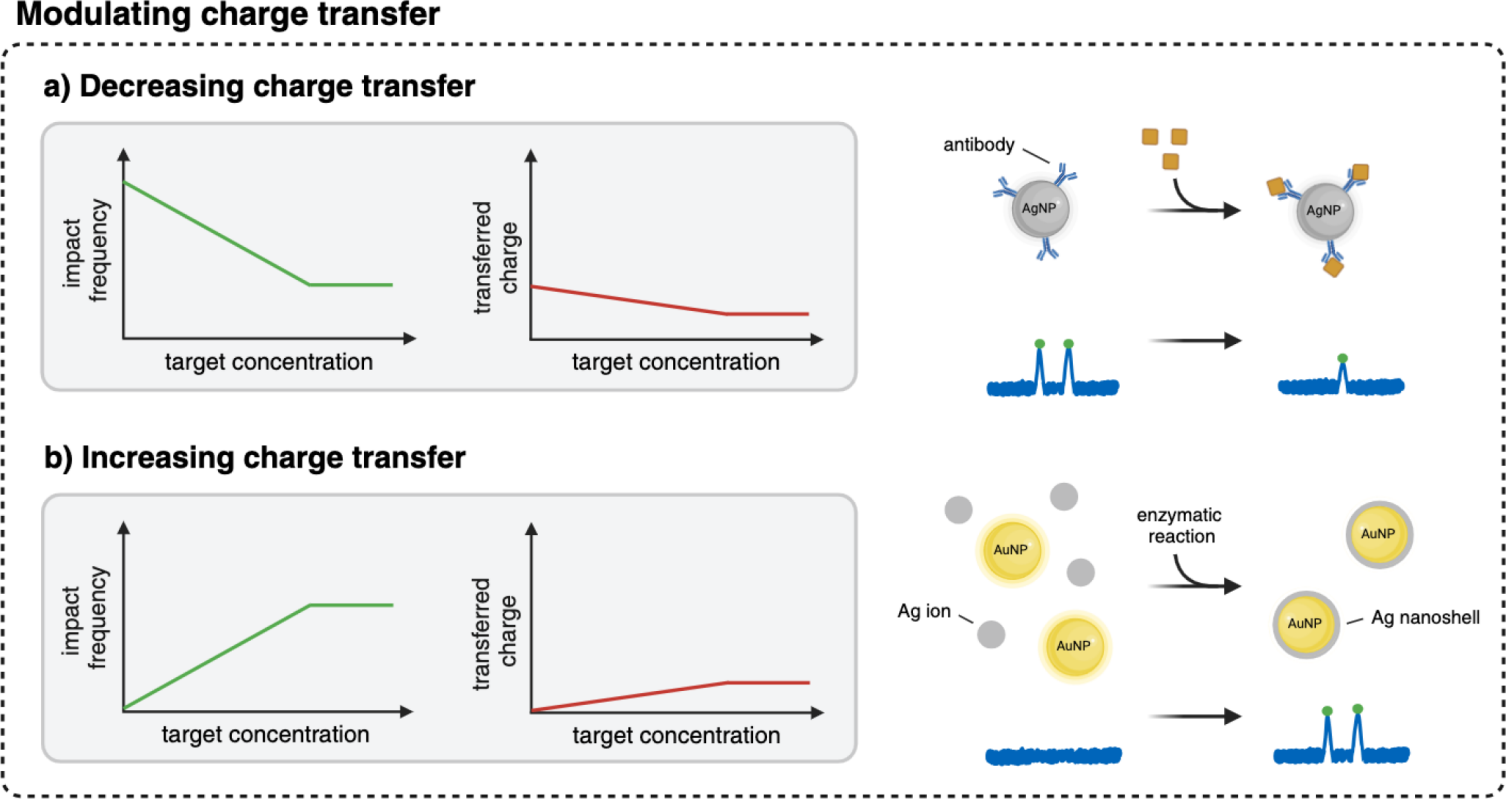
Representative strategies to modulate the charge transfer. (a) Increasing the corona thickness impairs the electron transfer from the AgNP to the electrode, leading to a decrease in impact frequency. (b) Deposition of Ag ions on AuNPs using an enzymatic reaction synthesizes a silver nanoshell, which can be oxidized. Consequently, the impact frequency and transferred charge increase with the target concentration. The *x*-axis (target concentration) often represents a logarithmic scale. This figure was created with BioRender.com.

**Table 1:** Summary of Reported Characteristics of Recent Biosensing Concepts Using Direct Collision Electrochemistry as a “Digital” Readout

strategy	target	AgNP size	electrode		detection conditions	reported range	LOD	ref.
			size (diameter)	material				
capture and release	biotin	40 nm	13 × 125 μm (62-MEA)	platinum	35 mM KCl, 50 mM KOH	10 pM–10 nM	10 pM	^59^
	HPV-16	20 nm	8 μm (62-MEA)	platinum	50 mM KOH, 200 mM KCl	40 pM–4 nM	25 pM	^45^
	miR-141	150 nm	3 mm	S–Au–SPE	50 mM KCl	10 aM–1 nM	4.21 aM	^53^
	SARS-CoV-2	43 ± 10 nm	25 μm	Ps-Au UMEs	10 mM Na_2_S_2_O_3_, 10 mM NaOH	10 fg/mL–1 ng/mL	6.78 fg/mL	^58^
	MCF-7 cells	47 ± 19 nm	25 μm	Ps-Au UMEs	10 mM Na_2_S_2_O_3_, 10 mM NaOH	5–10^5^ cells/mL	5 cells/mL	^57^
aggregation/deaggregation	alpha-fetoprotein	20 nm	7 μm	carbon fiber	10 mM KCl	5 pg/mL–500 ng/mL	5 pg/mL	^55^
	PFOS	28 nm	10 μm	carbon fiber	20 mM citrate buffer (pH = 6)	0.01–100 ppb	1 ppb	^60^
	ochratoxin A	30–40 nm	5 μm	carbon fiber	phosphate buffer (pH = 7.4)	-	0.05 nM	^103^
	proteinkinase A	20 nm	7 μm	carbon	10 mM KCl	0.01–0.10 U/μL	0.0048 U/μL	^56^
modulating charge transfer	CYFRA21-1	40–50 nm	4.9 μm	carbon fiber	10 mM KCl	0.1–10 ng/mL	0.1 ng/mL	^54^
	alkaline phosphatase	25 nm AuNPs	13.4 μm	gold	20 mM KCl	2–80 mU/mL	2 mU/mL	^104^

### Capture and Release of AgNPs

Direct single-impact electrochemistry can also be combined with typical ELISA strategies that capture the particles in the presence of the target molecules to establish an immunoassay with a “digital” readout (Figure 3a). In a sandwich-type assay, antibody-labeled AgNPs bind to immobilized antibodies in the presence of the target molecule, forming a captured complex. Unbound AgNPs remain in the solution and can be detected downstream at the electrodes. With an increasing target concentration, more AgNPs are captured, decreasing the impact frequency. Furthermore, the “digital” impact-based readout can be applied in a competitive binding setting, as demonstrated in a lateral flow assay designed for small molecule detection using biotin as a model system.^59^ The sensor comprised a 62-detection electrode chip integrated with a membrane-based microfluidics system. In this assay, biotinylated AgNPs compete with free sample biotin to bind to streptavidin-modified latex beads at the capture area. Unbound biotin-AgNPs pass the capture area and collide with the microelectrode array. As the target concentration increased, fewer biotinylated AgNPs were captured, resulting in a higher impact frequency. Although biotin is not a clinically relevant target, this “digital” lateral flow proof-of-concept highlights the potential for POC applications.

### Release of AgNPs

In contrast to capturing the particles, another approach to leverage AgNPs in direct collision electrochemistry involves the release of preimmobilized or stored particles, as schematically shown in Figure 3b. This strategy has been effectively combined with the clustered regularly interspaced short palindromic repeats (CRISPR)-based diagnostics. Certain CRISPR-associated (Cas) enzymes, such as Cas12 and Cas13, are characterized by a collateral cleavage activity that unfolds upon target recognition, leading to the nonspecific digestion of ssDNA or ssRNA. This property has been used to release immobilized DNA-AgNPs from MBs using Cas12a.^45^ As the collateral cleavage activity is a catalytic process with multiple turnovers, the number of released particles is determined by the concentration of the target and can be tuned using the incubation time with the activated Cas enzyme–the higher the target concentration and the longer the incubation time, the more particles are released and collide with the electrode. However, these systems are therefore sensitive to time and the Cas activity, which is known to be highly dependent on several factors, including temperature,^100^ ionic strength,^101^ and length of the activator.^102^ The combination of CRISPR-based diagnostics with direct impact electrochemistry shows potential for nucleic acid quantification at the POC. Furthermore, the Cas12 enzyme has been reported to release unmodified AgNPs stored in a DNA hydrogel, providing a label-free detection scheme.^53^ However, the concept has a longer sample-to-result time due to additional target amplification steps. In a different particle release approach, Wang and co-workers developed a method using PBST (phosphate-buffered saline containing 0.05% Tween-20) to release unmodified AgNPs stored in liposomes (Figure 3b).^57,58^ For SARS-CoV-2 detection, magnetic separation was used to isolate the liposomes containing AgNPs. The liposomes were designed to bind to MBs only in the presence of the target virus. Although the AgNPs in this approach were unfunctionalized, the concept could be applied using clinical samples, as the target recognition was separated from the AgNP detection. This allows the optimization of the detection conditions and prevents aggregation of unfunctionalized particles in complex media. In addition, DNAzyme-assisted signal amplification enhances the sensitivity by allowing more liposomes to bind than there are target molecules in the solution. By adding PBST buffer, the liposomes are hydrolyzed, releasing the AgNPs. The same release mechanism was used to quantify MCF-7 cells. Here, AgNP-containing liposomes were immobilized on antibody-modified MBs specific to MCF-7 cells. After magnetic separation, the AgNPs were released using PBST and detected on a polysulfide-modified gold ultramicroelectrode, which is also commonly used in other label-free approaches, as seen in Table 1. Alternatively, Luy et al. designed redox liposomes that release potassium ferrocyanide in the presence of Rhamnolipid toxin.^105^ A general benefit of the release-based approaches is the opportunity to couple the “digital” readout with self-amplifying release mechanisms, such as CRISPR/Cas or DNAzymes, to increase the number of released AgNPs per target molecule and reduce detection times. Furthermore, the impact frequency with this strategy would be zero when no target molecule is present, assuming an ideal system in which the particles are only released in the presence of the target molecule. This property could be used in combination with a high detection efficiency to achieve a very low limit of detection without target amplification.

### Aggregation/Deaggregation of AgNPs

As the aggregation of nanoparticles significantly alters their electrochemical properties, the aggregation and deaggregation of AgNPs induced by a target is a prominent strategy in direct collision electrochemistry. Aggregation leads to larger particles, resulting in different impact characteristics, including a decreased impact frequency and potentially an increased transferred charge (Figure 4a). Target-induced aggregation of the particles can be caused by specific recognition of labeled species on the AgNP surface, e.g., antibodies. Zhou and co-workers introduced such a strategy for detecting alpha-fetoprotein, a tumor protein biomarker.^55^ The mechanism relied on the aggregation of antibody-functionalized (anti-alpha-fetoprotein) AgNPs in the presence of alpha-fetoprotein. In this case, the thicker particle corona generally leads to lower peak amplitudes (Figure 2a) but simultaneously results in increased colloidal stability.

Particle aggregation can be likewise induced by species that directly adsorb onto unmodified AgNPs. Andreescu and co-workers introduced a label-free aggregation-based strategy to quantify per- and polyfluoroalkyl substances (PFASs), specifically perfluorooctanesulfonic acid (PFOS).^60^ The PFOS molecules are selectively adsorbed to the surface of unmodified AgNPs via the sulfonate group, inducing aggregation through F–F interaction between the PFOS molecules. Label-free induced aggregation typically results in larger peak amplitudes compared to the aggregation of labeled AgNPs due to the impaired electron transfer caused by the large corona in the latter. In addition, plain particles are more susceptible to changes in spike amplitude and charge transfer and they generally aggregate faster. However, the faster aggregation kinetics limit label-free methods to low electrolyte concentrations, which, in turn, is associated with lower impact frequencies (Figure 2c). Consequently, higher electrolyte concentrations–often found in biological samples, for example, in serum with a sodium concentration of around 140 mM^106^–could therefore lead to false positive results if target detection and impact readout are not separated.

In contrast to aggregation-based sensing, the deaggregation of AgNPs caused by a target increases the impact frequency until all particles are separated, as illustrated in Figure 4b. In 2016, Andreescu and co-workers introduced a deaggregation approach based on aptamer-functionalized AgNPs for the detection of Ochratoxin A.^103^ The aptamer sequences, partially complementary, caused the AgNPs to aggregate in absence of Ochratoxin A. Therefore, no impacts were observed possibly due to a high degree of aggregation. In the presence of Ochratoxin A, the aptamers captured the target, leading to the deaggregation of the functionalized particles and an increase in impact frequency. The transferred charge should decrease with increasing target concentration (Figure 4b), opposite to the aggregation-based strategies, as larger aggregates at low target concentrations are expected to transfer more charge per impact than individual particles. In the reported study, an increase in the impact frequency and a decrease in the estimated particle sizes based on the transferred charge were observed, indicating the successful deaggregation of the aptamer-functionalized particles in the presence of Ochratoxin A. In a different study, a label-free deaggregation method was demonstrated to detect and monitor the enzyme activity of protein kinase A.^56^ Here, a substrate peptide carrying two positive charges caused aggregation by screening or neutralizing the surface charges of the AgNPs. In the presence of protein kinase A and ATP, the substrate peptide displays a phosphate group with two negative charges, leading to deaggregation and an increased impact frequency.

### Modulation of Charge Transfer during AgNP Impacts

A further approach for biosensing via direct collision electrochemistry is based on the modulation of the charge transfer in the presence of the target species, as schematically illustrated in Figure 5. Increasing the corona thickness due to target binding to the AgNP impairs the electron transfer from the particle to the electrode (Figure 5a). In 2021, Zhang et al. introduced such an approach to detect CYFRA21-1, a tumor protein biomarker. This method is based on the decrease of impact frequency and transferred charge after AgNPs are modified with antibodies and consequently capture the target protein.^54^ With increasing CYFRA21-1 binding, both the impact frequency and the oxidative charge decreased. This electrochemical immunoassay was tested in diluted healthy serum spiked with the biomarker and clinical samples from lung cancer patients. The results were consistent with those obtained from traditional ELISA measurements, confirming the feasibility of direct collision electrochemistry as a “digital” readout to quantify protein-based biomarkers. Modulating the redox activity of particles is more commonly used in catalytically amplified strategies to detect nucleic acids, such as DNA and miRNA.^107–109^

In contrast, the charge transfer can also be increased by the deposition of Ag ions, e.g., using an alkaline phosphatase-catalyzed silver deposition reaction (Figure 5b). This strategy was used to synthesize a nanoshell on the surface of AuNPs, enabling the measurement of the alkaline phosphatase activity with a quantification limit of 2 mU/mL in 10 μL.^104^

## Conclusions and Outlook

In this review, we focused on recent advances in direct impact electrochemistry using AgNPs for biosensor applications. Additionally, we analyzed the key parameters influencing direct single-impact electrochemistry and their interconnections. Current strategies in direct collision electrochemistry still use electrodes that are predominantly below 25 μm in combination with particles mostly <50 nm, as summarized in Table 1. The use of arrays of individually addressable electrodes is beneficial for improving sensitivity, reducing the assay time, or parallel detection of multiple targets. However, fabricating microelectrode arrays to provide sufficiently low baseline noise is more challenging and expensive, as it typically requires a cleanroom environment, including photolithography, material deposition, and selective etching techniques. For this reason, future research should prioritize the use of larger nanoparticles that undergo oxidation with significant charge transfer. Such an approach could simplify detection through higher spike amplitudes detectable using larger electrodes with less complex and low-cost electronics. In addition, larger electrodes can be fabricated using rapid prototyping techniques, such as laser patterning, increasing throughput and decreasing the costs of devices. By employing smart electrode designs and fabrication strategies–for example, using foils for passivation–cleanroom processes can largely be avoided, thereby enhancing the suitability of the chips for POC applications. Alternatively, larger nanoparticles can also be used to amplify the signal, as one target molecule results in multiple spikes. Furthermore, future research efforts should focus on enhancing the detection efficiency by improving the mass transport to the electrode, as it is still a major limitation of the current concepts, particularly from strategies that solely rely on diffusion. Promising approaches could include the integration of (paper-based) microfluidics, such as lateral flow concepts, three-dimensional electrode arrays, and magnetophoresis with AgNPs featuring a magnetic core. Additionally, novel concepts should be developed to apply the readout in biological samples without impacting the particle properties. This can, for example, be achieved by optimizing electrolyte conditions and separating the target recognition phase and the AgNP detection phase. Such an approach will allow the optimization of the detection conditions without reducing interference from the sample solution. Moreover, a benefit of measuring discrete signals is the rapid and straightforward analysis of the data.^110^ Nevertheless, future high-density electrode detection arrays, for instance, based on complementary metal oxide semiconductor systems and, consequently, large data sets, will benefit from current developments in data processing techniques supported by artificial intelligence.^94,111^ A recent review discusses emerging data processing methods for single-entity electrochemistry.^94^ Finally, addressing further challenges, such as signal reproducibility and nanoparticle stability, will be crucial for advancing this technology. A future POC chip should be robust enough to be stored for several weeks without impacting the readout signal, e.g., by drying the AgNPs on the chip. Additionally, *in situ* monitoring of the landing of individual particles from solution onto the detection electrodes would promote a better understanding of collision-based electrochemistry. By offering a comprehensive overview of the influencing factors and their implications, alongside an analysis of recent advances, we aim to facilitate the design of novel nanoimpact electrochemical platforms, particularly for POC applications. This should open the field of electrochemical impact-based biosensing to other researchers and accelerate future developments. The “digital” readout based on direct-impact electrochemistry could eventually extend the capability of existing diagnostic approaches, enabling not only target detection but also quantification, such as in classical lateral flow immune-based assays. Furthermore, integrating nanoparticle impact detection with isothermal nucleic acid amplification–such as recombinase polymerase amplification or loop-mediated isothermal amplification–could pave the way for low-cost POC nucleic acid quantification with similar detection limits as gold standard lab-based techniques, such as polymerase chain reaction.

## Abbreviations

AgNP: silver nanoparticle
AuNP: gold nanoparticle
POC: point-of-care
PtNPs: platinum nanoparticles
SNR: signal-to-noise ratio
PFOS: perfluorooctanesulfonic acid
CRISPR: clustered regularly interspaced short palindromic repeats
PBST: phosphate-buffered saline containing Tween
MEA: microelectrode array
MB: magnetic bead

## Vocabulary Section

impact electrochemistry: Also referred to as collision electrochemistry: An analytical method that is based on the collision of single entities, such as nanoparticles, with a biased microelectrode.
“digital” readout: A sensing approach where discrete current spikes (“1”) compared to the background noise (“0”) enable a binary-like quantification readout of target molecules.
current spike: A transient sharp increase/decrease in current observed when a single nanoparticle collides with an appropriately biased electrode, typically associated with redox reactions or capacitive effects.
impact frequency: The rate at which nanoparticles collide with an electrode within a defined measurement time, which can be correlated to the concentration of the target analyte
direct impact: Collision of redox-active species, such as silver nanoparticles, with a microelectrode, resulting in a distinct current spike due to partial or complete oxidation of the species.
